# Development of genome-wide polymorphic microsatellite markers for *Trichinella spiralis*

**DOI:** 10.1186/s13071-020-3929-2

**Published:** 2020-02-11

**Authors:** Ting-Ting Li, Bin Tang, Xue Bai, Xue-Lin Wang, Xue-Nong Luo, Hong-Bin Yan, Hong-Fei Zhu, Hong Jia, Xiao-Lei Liu, Ming-Yuan Liu

**Affiliations:** 1 Key Laboratory of Zoonosis Research, Ministry of Education, Institute of Zoonosis, College of Veterinary Medicine, Jilin University, Changchun, 130062 Jilin People’s Republic of China; 2 State Key Laboratory of Veterinary Etiological Biology, Key Laboratory of Veterinary Parasitology of Gansu Province, Lanzhou Veterinary Research Institute, CAAS, Lanzhou, 730046 Gansu People’s Republic of China; 3 Institute of Animal Sciences, Chinese Academy of Agricultural Sciences, Beijing, 100193 People’s Republic of China; 4 Jiangsu Co-innovation Center for Prevention and Control of Important Animal Infectious Diseases and Zoonoses, Yangzhou, Jiangsu People’s Republic of China

**Keywords:** *Trichinella spiralis*, Microsatellite, Cross-amplification

## Abstract

**Background:**

*Trichinella* nematodes are globally distributed food-borne pathogens, in which *Trichinella spiralis* is the most common species in China. Microsatellites are a powerful tool in population genetics and phylogeographic analysis. However, only a few microsatellite markers were reported in *T. spiralis*. Thus, there is a need to develop and validate genome-wide microsatellite markers for *T. spiralis*.

**Methods:**

Microsatellites were selected from shotgun genomic sequences using MIcroSAtellite identification tool (MISA). The identified markers were validated in 12 isolates of *T. spiralis* in China.

**Results:**

A total of 93,140 microsatellites were identified by MISA from 9267 contigs in *T. spiralis* genome sequences, in which 16 polymorphic loci were selected for validation by PCR with single larvae from 12 isolates of *T. spiralis* in China. There were 7–19 alleles per locus (average 11.25 alleles per locus). The observed heterozygosity (*H*_*O*_) and expected heterozygosity (*H*_*E*_) ranged from 0.325 to 0.750 and 0.737 to 0.918, respectively. The polymorphism information content (PIC) ranged from 0.719 to 0.978 (average 0.826). Among the 16 loci, markers for 10 loci could be amplified from all 12 international standard strains of *Trichinella* spp.

**Conclusions:**

Sixteen highly polymorphic markers were selected and validated for *T. spiralis*. Primary phylogenetic analysis showed that these markers might serve as a useful tool for genetic studies of *Trichinella* parasites.
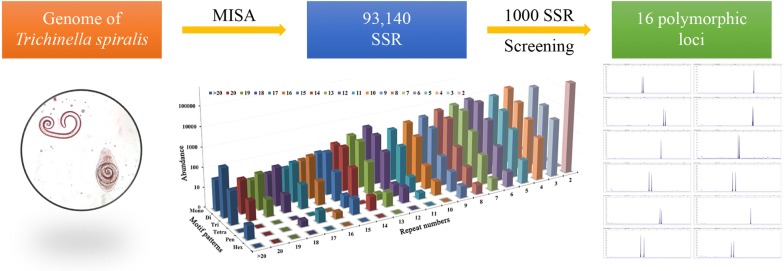

## Background

Human trichinellosis is caused by eating raw or undercooked meat infected with *Trichinella* parasites [1]. *Trichinella* parasites have a broad geographical distribution on all continents except Antarctica, and can infect > 150 animal species, including mammals, birds and reptiles [2]. The genus *Trichinella* contains nine species and three genotypes that can be separated into two clades by the ability to form encapsulated and non-encapsulated larvae [3–5]. There are genetic variations in *Trichinella* spp. based on geographical distributions and host species [6, 7]. In China, *Trichinella* spp. have been reported in a range of animals, including foxes, bears, wild boar, weasels, raccoon dogs, rats, bamboo rats and civets [8]. Only two *Trichinella* species (i.e. *T. spiralis* and *T. nativa*) have been identified in China [8–12]. However, little is known about the genetic variations among the *Trichinella* species in China.

Genetic variability in *T. spiralis* was first reported in 1992, with three allozyme patterns at the loci of glucose 6-phosphate dehydrogenase and glucose phosphate isomerase detected in 61 isolates of *T. spiralis* from zoogeographical regions [6]. Genetic polymorphisms in *T. spiralis* were also studied using different molecular tools, such as restriction fragment length polymorphism and single-strand conformational polymorphism (RFLP-SSCP) [13, 14], non-isotopic single-strand conformation polymorphism (‘cold’ SSCP) [15], and deep resequencing of the mitochondrial genomes [16]. Compared with other molecular markers, microsatellites exist throughout the genome. In addition, microsatellites are relatively easy to score, since their gel band patterns could provide unambiguous results. Thus, they have been widely used in genetic diversity, population genetic structure, genome mapping, parentage analysis, population genetics and phylogeography studies [17–19]. However, only a few microsatellites have been reported in *T. spiralis* [12, 20–22]. The present study was aimed to identify and characterize microsatellites in *T. spiralis* and to obtain polymorphic microsatellite markers for further study.

## Methods

### Parasites

**Fig. 1:**
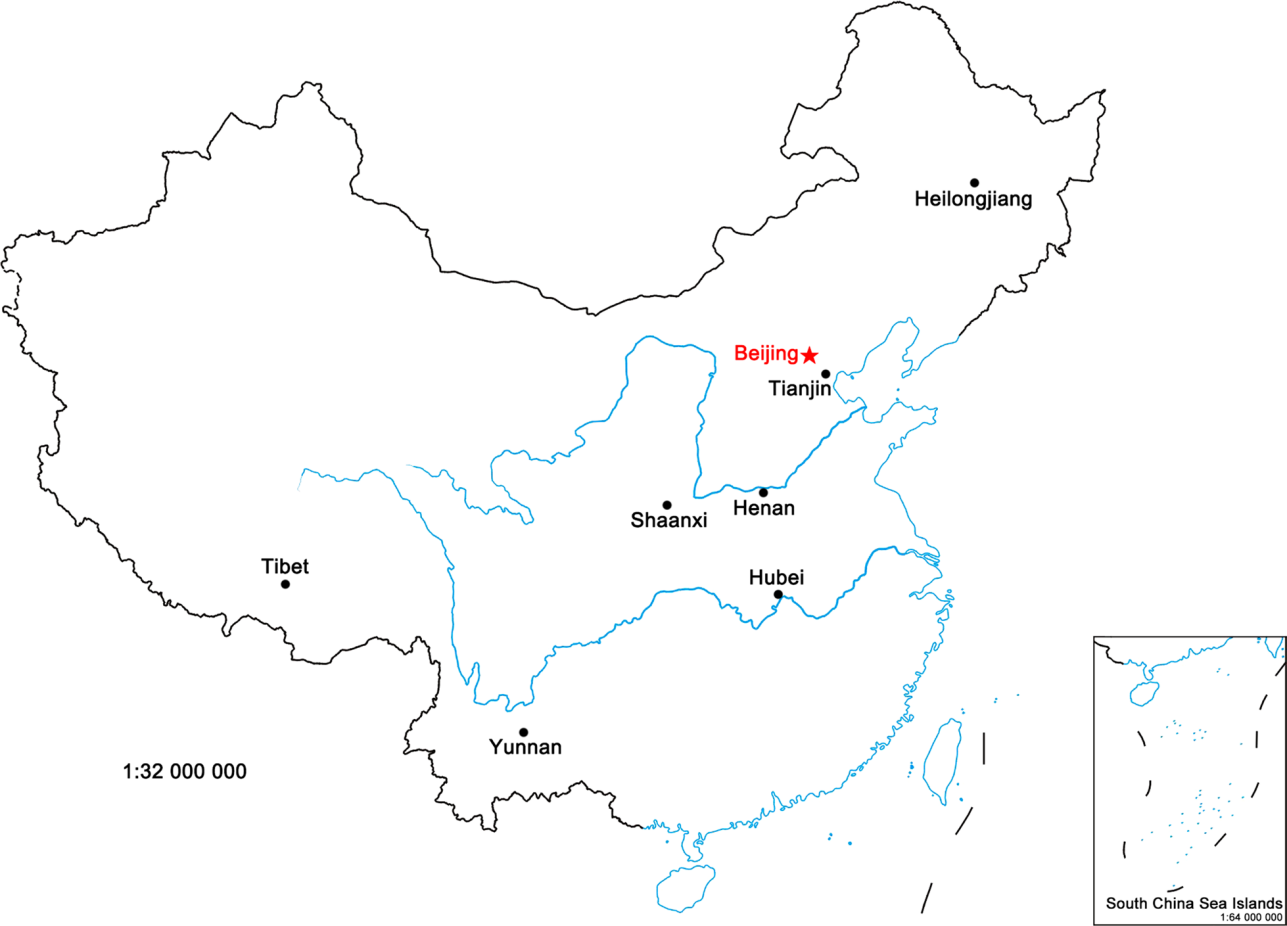
Distribution of *Trichinella spiralis* isolates from different geographical regions in China. Twelve isolates of *T. spiralis* were obtained from seven regions in China: five from Tianjin city; two from Yunnan Province; and one each from Heilongjiang, Henan, Hubei, Shaanxi and Tibet, respectively

Twelve isolates of *T. spiralis* were obtained from seven regions in China: five from Tianjin city, two from Yunnan Province, and one each from Heilongjiang, Henan, Hubei, Shaanxi and Tibet, respectively (Fig. 1). All isolates were confirmed as *T. spiralis* using multiplex PCR method according to Zarlenga et al. [23]. The following 15 international standard *Trichinella* strains were acquired from the International *Trichinella* Reference Centre (ITRC; Rome, Italy): *T. spiralis* (T1, ISS534 and ISS4); *T. nativa* (T2, ISS70); *T. britovi* (T3, ISS100); *T. pseudospiralis* (T4, ISS13, ISS141 and ISS470); *T. murrelli* (T5, ISS415); *Trichinella* T6 (ISS34); *T. nelsoni* (T7, ISS37); *Trichinella* T8 (ISS124); *Trichinella* T9 (ISS408); *T. papuae* (T10, ISS572); *T. zimbabwensis* (T11, ISS1029); and *T. patagoniensis* (T12, ISS1826). All isolates and strains were maintained by serial passages in ICR mice. Larvae were recovered from the muscle tissues of infected mice on day 35 post-infection by an artificial digestion method [24], and stored at − 80 °C until use.

### Microsatellite identification and primer design

All 9267 contigs of *T. spiralis* were retrieved from GenBank database (https://www.ncbi.nlm.nih.gov/nuccore/ABIR00000000) and used to search for microsatellite sequences by MIcroSAtellite Identification Tool (MISA) that was configured with strict minimum motif repeat requirements [25]. The criteria of motifs were that mono- to hexanucleotide repeats with a minimum of 12 bp and a minimum of two repeat units. The maximum length of sequence between two simple sequence repeats (SSRs) to register as compound SSR was 100 bp [19]. The number of microsatellites, motif, number of repeats, length of the repeat sequence, repeat type, start and end position of the repeat sequence, and microsatellite sequence, were analyzed using MISA.

Primers flanking the putative microsatellite sequences were designed at the PRIMER3 online server (http://primer3.ut.ee) [26], using following parameters: optimal primer length = 20 bp (between 18–22 bp); optimal primer GC content = 50% (between 40–60%); optimal primer melting temperature = 58 °C (between 55.9–60.1 °C); and product size ranged from 150 to 300 bp. The melting temperatures between a pair of primer had < 1 °C difference. The specificity of primer sequences was determined by BLAST searches against the genome of *T. spiralis* (https://www.ncbi.nlm.nih.gov/tools/primer-blast/).

### Screening of microsatellites by PCR

A total of 1000 SSR primer pairs were selected for preliminary screening by PCR using DNA from a pool of ~ 4000 muscle larvae (~ 350 larvae from each of the 12 *T. spiralis* isolates in China). For isolating DNA, all larvae were homogenized in 500 μl extraction buffer containing 500 mM NaCl, 10 mM Tris-Cl (pH 8.0), 50 mM EDTA (pH 8.0), 2% (w/v) SDS and 10 mM β-mercaptoethanol, followed by incubation with 5 μl of proteinase K (20 mg/ml) at 60 °C for 0.5–2 h, phenol-chloroform extraction (50:50%, v/v), precipitation with 70% ethanol, and resuspension in 30–50 μl of sterile water. DNA samples were stored at − 20 °C. PCR reactions were carried out in a final volume of 20 μl, consisting of ~ 50 ng of DNA, 2 μl of 10× Ex Taq buffer (20 mM Mg^2+^ Plus; TaKaRa, Kusatsu, Japan), 1.6 μl of dNTP mixture (2.5 mM each), 0.2 μl of Ex Taq DNA polymerase (5 U/μl) (TaKaRa), and 0.4 μl of each primer (10 pmol/μl). PCR amplifications were performed in a thermal cycler (Applied Biosystems, California, USA) using following program: 98 °C for 5 min; followed by 35 cycles of 98 °C for 10 s, a specified annealing temperature for each primer pair for 30 s, 72 °C for 30 s; and a final extension step at 72 °C for 7 min. PCR products were electrophoresed on 1% agarose gels, stained with ethidium bromide and visualized under UV illumination. Microsatellite markers producing single bands were selected as candidate loci for further validation.

### Verification of microsatellite polymorphism

Each of the selected primers was validated with 40 single larvae of *T. spiralis* from seven regions in China. Single larva was digested with proteinase K for DNA extraction using a Tissue and Hair Extraction Kit and a DNA IQ™ System Extraction Kit (Promega, Madison, USA) with magnetic beads following manufacturer’s instructions. DNA was eluted in 25 μl of elution buffer. Whole genome amplification was performed using an Illustra™ Ready-To-Go™ GenomiPhi V3 DNA Amplification Kit (GE Healthcare, Pittsburgh, USA) to increase the quantity of DNA. Concentrations of DNA were measured in a NanoDrop 2000 photometer (Thermo Fisher Scientific, Waltham, USA).

PCR amplifications were performed in a 20 μl reaction using a primer mixture which contained three primers: a sequence-specific forward primer with M13-tail at its 5′-end, a sequence-specific reverse primer, and the universal fluorescent-labeled M13 primer (FAM-M13 primer) [27]. A 20 μl reaction contained 0.05 μM forward primer, 0.25 μM reverse primer, 0.2 μM FAM-M13 primer, 0.16 mM dNTP, 1 U of Ex Taq DNA polymerase (TaKaRa), and ~ 50 ng of DNA from a single larva [27]. The PCR program was run as follows: 98 °C for 5 min; 32 cycles of 98 °C for 10 s, an annealing temperature specified for a primer pair for 30 s, and 72 °C for 30 s; eight additional cycles of 98 °C for 10 s, 53 °C for 30 s and 72 °C for 30 s; a final extension at 72 °C for 7 min. PCR products were subjected to capillary electrophoresis analysis (CEA) with a 96-capillary 3730XL DNA Analyzer (Applied Biosystems). Data were analyzed with GeneMapper 4.0 (Applied Biosystems). A negative control with sterile water was included in each PCR run.

Finally, the microsatellite loci with high polymorphism were selected for further validation by PCR using DNA samples isolated from individual larvae from 12 isolates of *T. spiralis* in China (10 larvae per isolate; total 120 samples). PCR amplification and analysis followed the protocols described above.

### Polymorphism analysis

For each locus, the number of alleles (*N*_*a*_), the effective number of alleles (*N*_*e*_), the expected heterozygosity (*H*_*E*_) and the observed heterozygosity (*H*_*O*_) per locus were estimated using GENEPOP version 4.2 (http://genepop.curtin.edu.au/) [28]. This same software was used to test the polymorphism information content (PIC) and possible deviations from Hardy–Weinberg equilibrium (HWE) with Bonferroni correction [29].

### Cross-amplification

DNA samples were isolated from the 12 *Trichinella* international standard strains as described in section “Screening of microsatellites by PCR” above. Cross-amplifications at selected polymorphic loci were performed and analyzed by a capillary electrophoresis using the same PCR protocols as described in section “Verification of microsatellite polymorphism” above.

### Phylogenetic analysis

The PCR products amplified from 15 international standard strains at the TsMs03 locus were analyzed by 8% denaturing urea-polyacrylamide gel electrophoresis. The homozygous individuals were selected for sequencing. Multiple sequence alignments of nucleotide sequences at the TsMs03 locus were performed using Clustal Omega (https://www.ebi.ac.uk/Tools/msa/clustalo/) [30]. The phylogenetic tree was inferred by MEGA X using the Neighbor-Joining method with 1000 bootstrap replicates [31, 32].

## Results

### Abundance and microsatellite characteristics

**Fig. 2:**
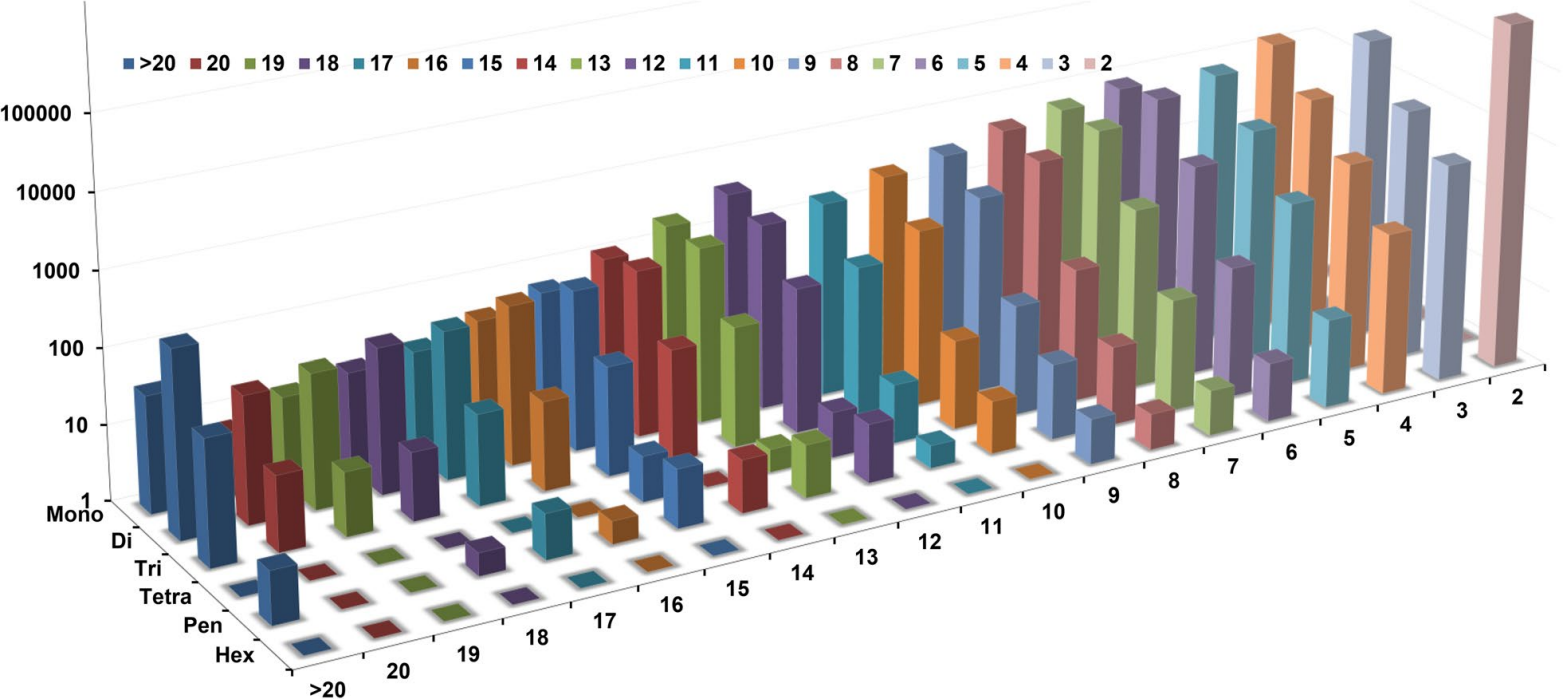
Distribution in relation to the microsatellite repeat number of mono- to hexanucleotide motifs in the whole genome sequences of *Trichinella spiralis*. The vertical axis shows the abundances of microsatellites that have different motif repeat numbers (from 2 to > 20), which are discriminated by the legends of different colors

**Fig. 3:**
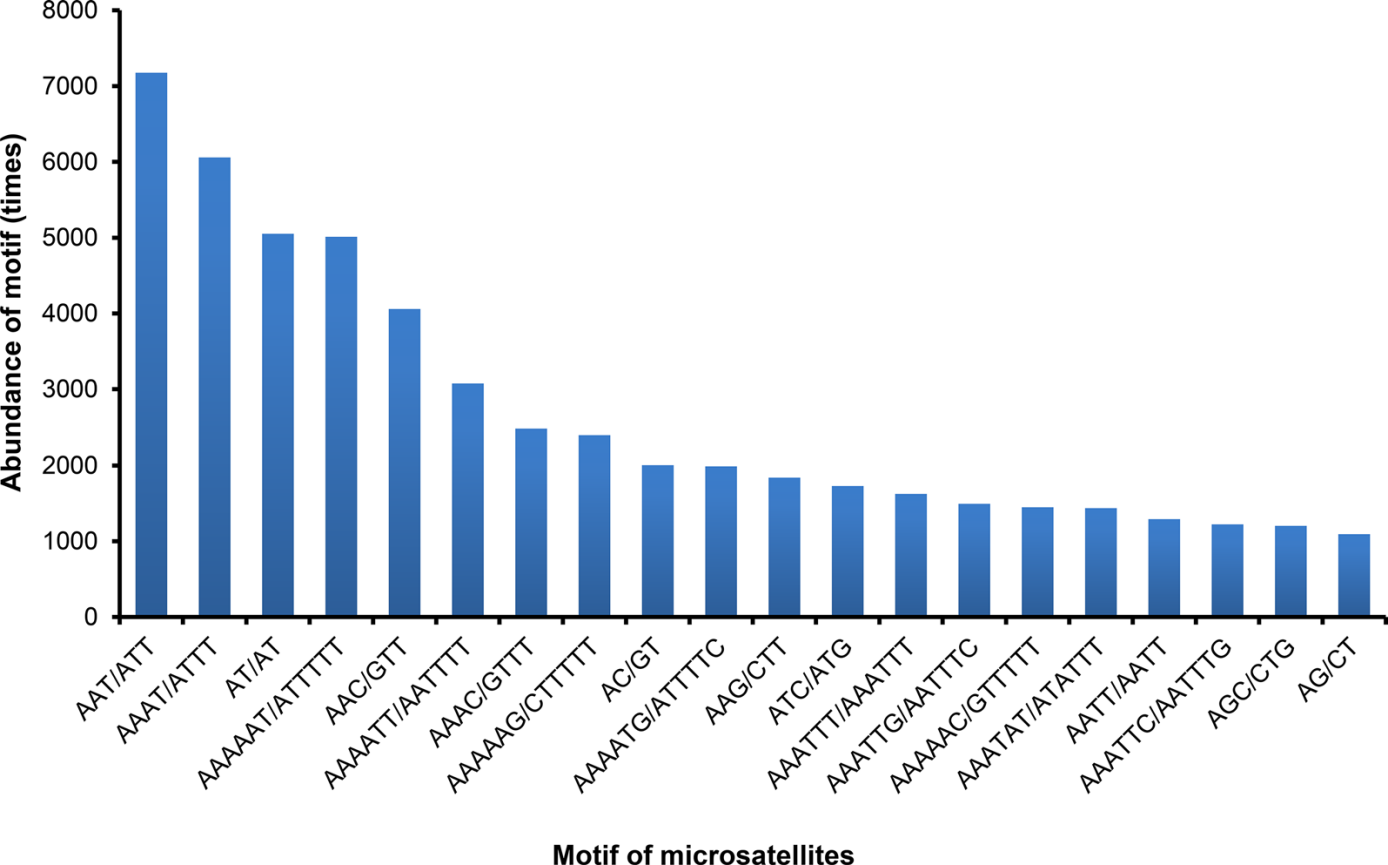
The 20 most frequently classified repeat types (considering sequence complementary) in *Trichinella spiralis*. The most common motifs in each type of repeats were A/T, AT/AT, AAT/ATT, AAAT/ATTT, AAAAT/ATTTT and AAAAAT/ATTTTT

A total of 93,140 microsatellites were identified from 9267 contigs of the *T. spiralis* genome by MISA (Table 1). The microsatellite density was 1591 loci per Mb. Among motifs containing mono- to hexanucleotide repeats, the most abundant was hexanucleotides that accounted for 49.51% of the total, followed by trinucleotide (19.61%) and tetranucleotide (17.44%). The di-, penta-, and mononucleotide motifs accounted for 8.77%, 3.69%, and 0.98% of the total motifs, respectively. The significant decrease in abundance of microsatellites was accompanied by the increase in the number of motif repeats. The number of repeating nucleotide sets was two times in 97.81% of hexanucleotide repeats. Meanwhile the number was three times in 1.81% of hexanucleotide repeats. For the pentanucleotide repeats, 68.29% consisted of three repeats, 19.12% consisted of four repeats, 8.18% consisted of five repeats, and 1.63% consisted of six repeats (Fig. 2). The top 20 most frequently classified repeat types were listed in Fig. 3. The most common motifs in each type of repeats were A/T (59.43%), AT/AT (61.84%), AAT/ATT (39.28%), AAAT/ATTT (37.30%), AAAAT/ATTTT (18.07%) and AAAAAT/ATTTTT (10.87%). The longest repeat was (TATAA)_98_ which belonged to the pentanucleotide group (Table 2).

**Table 1:** Motif statistic of *Trichinella spiralis* microsatellites

Motif	Total counts	Distribution (%)	Average length	Counts/Mbp
Mononucleotide	912	0.98	14.28	0.013
Dinucleotide	8166	8.77	18.71	0.152
Trinucleotide	18,267	19.61	16.23	0.297
Tetranucleotide	16,241	17.44	14.25	0.231
Pentanucleotide	3437	3.69	18.3	0.063
Hexanucleotide	46,117	49.51	12.16	0.561

**Table 2:** Most common and the longest microsatellites of the motifs

Repeat	Mononucleotide		Dinucleotide		Trinucleotide		Tetranucleotide		Pentanucleotide		Hexanucleotide	
	Motif	%	Motif	%	Motif	%	Motif	%	Motif	%	Motif	%
Common	A/T	59.43	AT/AT	61.84	AAT/ATT	39.28	AAAT/ATTT	37.30	AAAAT/ATTTT	18.07	AAAAAT/ATTTTT	10.87
	C/G	40.57	AC/GT	24.53	AAC/GTT	22.21	AAAC/GTTT	15.27	AAATT/AATTT	10.53	AAAATT/AATTTT	6.67
			AG/CT	13.38	AAG/CTT	10.06	AATT/AATT	7.95	AATAT/ATATT	9.72	AAAAAG/CTTTTT	5.19
			CG/CG	0.24	ATC/ATG	9.46	AATG/ATTC	6.13	AAAAC/GTTTT	7.97	AAAATG/ATTTTC	4.31
					AGC/CTG	6.58	ACAT/ATGT	4.93	AATAC/ATTGT	7.22	AAATTT/AAATTT	3.52
					Other	12.40	Other	28.42	Other	46.49	Other	69.45
Longest	(G)_184_		(TG)_54_		(ATA)_64_		(CATA)_17_		(TATAA)_98_		(AATAGT)_9_ (TGTATA)_9_ (TATATG)_9_ (ATATAC)_9_	

### Polymorphic microsatellite screening

Among the 1000 microsatellite loci selected for primary screening, 676 loci generated PCR products at expected sizes. A total of 120 loci producing single bright band in gel electrophoresis were selected as candidate loci. Among them, 47 microsatellite loci were homozygotes, while 57 loci showed low polymorphism. Finally, we selected 16 loci that produced distinct bands among individual larvae originated from different regions in China with high polymorphism for further analysis (Table 3).

**Table 3:** Characteristics of 16 microsatellites and primer sets

Name	Primer sequence (5′-3′)	Repeat motif	Product size (bp)	Contig/ID	Position	Tm (°C)
TsMs01	F: GGGCATATATTACGCATACCG R: ACGACGAAATGATTCTTGCC	(TG)_25_	265–297	gi316972836	40205–40481	58
TsMs02	F: GATTGGGCAAAGGATGAATG R: AAAACGACGGCAAATCAAAC	(TTTG)_9_	157–177	gi316972836	18217–18374	58
TsMs03	F: TGTTACTTCATGTGGCAGAGTG R: GCCAACTGGATTTTAATGACAGA	(TAATT)_17_	221–297	gi316972363	130723–131001	60
TsMs04	F: CTAAGGCATCGCTGGTTTTT R: TGATTGGCTATCAAGCAACG	(ATC)_11_	246–275	gi316973090	19425–19675	58
TsMs05	F: CGACAACTTCAACGACGGTA R: TCGCTTCATCAGAGGGAACT	(GTTT)_9_	260–292	gi316969813	186555–186801	60
TsMs06	F: TAATGCTGGTTTGCGCTATG R: AACTGAGCGGAAATTTTGACA	(TAA)_10_	210–302	gi316973625	143441–143668	60
TsMs07	F: GGCCGTTTTGAAATGAAAAAT R: GCGTTGATTCAGCTAAGCGT	(ATA)_9_	252–276	gi316976532	119918–120196	60
TsMs08	F: GGGTGTCGTTGTCATTTGTG R: GGTGCGTGGAAATTGAAAAT	(TAG)_11_	259–290	gi316978307	145640–145883	58
TsMs09	F: CCTGCGGTTATTGTTTGCTT R: AGCCGGAGAGAATATGGGAT	(GTA)_9_(GTT)_8_	275–298	gi316978154	85694–85961	58
TsMs10	F: ACAGCCCATATTTTTCGACG R: CCAATTTTAAGCACATTGCG	(TAACA)_6_	212–245	gi316979296	17474–17750	60
TsMs11	F: GGATAGCACGTATTGGCGAT R: TTCAATGCTTTTCGATGCAG	(ACACAT)_6_	167–197	gi316978262	56111–56387	58
TsMs12	F: TGGAACAAATGCCATTCAAA R: CCCTGAGCGCAATGTAAAGT	(AAG)_11_(ATG)_5_	210–226	gi316969236	19887–20088	58
TsMs13	F: GGTAAATGAGGTTCGCGTTC R: AGGATGTTATTCGCCCAGAA	(ATAA)_8_	213–272	gi316967561	66833–67009	60
TsMs14	F: TCCTGACCCAGTCCATTGAT R: AAATCGATAAGCATTTGGCG	(CTT)_8_	210–226	gi316977317	111618–111824	58
TsMs15	F: CCTACGCGATCAAGTGTTCA R: CTGCGTTTGTCCTCTGTTCA	(TTTG)_7_	213–272	gi316971889	83295–83500	56
TsMs16	F: GCCACCAGAGTGGACAAAAT R: GCGTTGAGTGAAGTGATGGA	(TAT)_22_	215–245	gi316977492	66935–67190	60

### Polymorphism analysis

*N*_*a*_ varied from 7 to 19, and *N*_*e*_ ranged from 5.655 to 14.452 (average 8.820) per locus. *H*_*O*_ and *H*_*E*_ ranged from 0.325 to 0.750 and 0.737 to 0.918, respectively. PIC ranged from 0.719 to 0.978 (average of 0.826). The final set of 16 microsatellite markers were all highly informative (PIC > 0.50), and four of the 16 loci showed significant deviations from HWE after Bonferroni correction (Table 4).

**Table 4:** Microsatellite markers and their polymorphism characteristics

Locus	*N*_*a*_	*N*_*e*_	*H*_*O*_	*H*_*E*_	PIC
TsMs01	11	7.42	0.325	0.857	0.845
TsMs02	11	7.923	0.675	0.785	0.719
TsMs03	19	14.452	0.75	0.918	0.978
TsMs04	12	9.901	0.65	0.895	0.843*
TsMs05	8	6.877	0.325	0.866	0.814*
TsMs06	7	5.655	0.55	0.831	0.733*
TsMs07	10	7.865	0.575	0.877	0.837
TsMs08	8	6.667	0.525	0.813	0.754
TsMs09	13	10.321	0.675	0.875	0.887
TsMs10	10	7.393	0.45	0.863	0.868*
TsMs11	11	8.542	0.575	0.857	0.821
TsMs12	16	12.279	0.475	0.924	0.815
TsMs13	9	6.957	0.625	0.874	0.834
TsMs14	10	8.733	0.675	0.862	0.804
TsMs15	8	6.641	0.575	0.737	0.736
TsMs16	17	13.476	0.55	0.943	0.925
Mean	11.25	8.82	0.561	0.861	0.826

*Abbreviations*: *N*_*a*_, observed number of alleles; *N*_*e*_, effective number of alleles; *H*_*E*_, expected heterozygosity; *H*_*O*_, observed heterozygosity; PIC, polymorphism information content

*Significant deviation from HWE after Bonferroni correction

### Cross-amplification

Among the final 16 loci, 10 produced PCR amplicons for all tested *Trichinella* spp. Four (i.e. TsMs01, TsMs04, TsMs10 and TsMs14) obtained PCR products only from the *Trichinella* spp. with encapsulated larvae. Most of these loci were homozygous in the *T. britovi* (encapsulated larvae) and species with non-encapsulated larvae (Table 5). In addition, the TsMs07 and TsMs08 loci were amplified from species with encapsulated and non-encapsulated larvae, except for *T. pseudospiralis*. The average number of amplified alleles in each of the *Trichinella* spp. ranged from 1.300 (*T. papuae* and *T. zimbabwensis*) to 2.938 (*Trichinella* T9). A maximum of six alleles was observed in *Trichinella* T9 strain at the TsMs03 locus. Allelic size varied among taxa at a given locus, and one allele was shared by two or three taxa commonly. *Trichinella* T9 had specific alleles at three loci (i.e. TsMs12, TsMs14 and TsMs16) that were different in allelic size from other *Trichinella* taxa. None of the alleles at a given locus were shared by all *Trichinella* spp.

**Table 5:** Cross-amplifications at 16 polymorphic loci in *Trichinella* spp.

Locus/taxa	*T. spiralis* ISS4		*T. nativa* ISS70		*T. britovi* ISS100		*T. pseudospiralis* ISS13		*T. murrelli* ISS415		*Trichinella* T6 ISS34	
	*N*_*a*_	Size (bp)	*N*_*a*_	Size (bp)	*N*_*a*_	Size (bp)	*N*_*a*_	Size (bp)	*N*_*a*_	Size (bp)	*N*_*a*_	Size (bp)
TsMs01	2	267–279	2	265–278	2	271–281	–	–	2	286–296	3	265–281
TsMs02	1	159	1	167	3	167–174	1	170	1	178	2	165–174
TsMs03	4	243–284	2	222–248	1	222	1	236	4	263–302	3	222–248
TsMs04	2	262–268	1	258	1	261	–	–	2	264–270	1	261
TsMs05	1	267	1	279	2	259–263	2	288–292	2	263–267	1	263
TsMs06	2	237–246	2	233–239	1	236	2	283–303	2	239–248	2	236–239
TsMs07	2	297–303	3	294–306	1	294	–	–	2	296–302	2	294–297
TsMs08	2	262–265	2	252–258	1	261	–	–	2	264–267	2	252–261
TsMs09	1	288	2	273–291	1	276	1	286	1	288	2	276–291
TsMs10	2	283–294	1	298	1	293	–	–	2	284–294	2	293–299
TsMs11	2	295–301	2	284–290	2	284–291	1	298	3	290–302	3	284–296
TsMs12	1	215	2	220–230	1	212	2	216–247	1	218	2	211–226
TsMs13	1	193	1	189	2	181–185	1	213	1	185	1	189
TsMs14	1	224	2	210–222	1	219	–	–	1	226	3	210–220
TsMs15	1	226	1	213	1	213	1	220	2	213–217	1	217
TsMs16	3	264–270	1	231	3	190–234	2	195–203	2	190–265	3	190–231

Locus/taxa	*T. nelsoni* ISS37		*Trichinella* T8 ISS124		*Trichinella* T9 ISS408		*T. papuae* ISS572		*T. zimbabwensis* ISS1029		*T. patagoniensis* ISS1826	
	*N*_*a*_	Size (bp)	*N*_*a*_	Size (bp)	*N*_*a*_	Size (bp)	*N*_*a*_	Size (bp)	*N*_*a*_	Size (bp)	*N*_*a*_	Size (bp)
TsMs01	3	265–302	3	265–302	3	269–289	–	–	–	–	2	286–296
TsMs02	3	158–179	2	158–165	4	158–175	2	158–170	1	158	1	178
TsMs03	5	222–267	5	222–258	6	222–252	2	241–247	1	241	4	262–301
TsMs04	3	258–267	2	247–261	3	255–261	–	–	–	–	2	264–270
TsMs05	1	259	1	263	1	259	1	272	1	276	2	267–271
TsMs06	2	236–239	2	236–242	2	236–242	1	225	2	224–256	3	239–248
TsMs07	3	285–306	2	288–294	2	288–294	1	285	2	296–302	2	284–293
TsMs08	3	252–261	3	252–261	3	255–264	2	252–258	2	261–264	2	264–267
TsMs09	2	273–282	3	270–288	2	273–282	1	280	1	272	1	288
TsMs10	3	284–298	3	275–298	4	284–304	–	–	–	–	2	284–293
TsMs11	4	281–296	4	284–297	4	278–288	2	286–296	2	286–296	2	296–302
TsMs12	3	215–237	3	211–242	4	215–240	1	217	1	248	1	218
TsMs13	1	177	1	177	1	185	1	207	1	207	1	185
TsMs14	2	210–220	2	210–220	2	214–220	–	–	–	–	1	226
TsMs15	1	225	1	217	1	213	1	219	1	210	1	224
TsMs16	2	190–222	3	190–234	5	190–245	1	191	2	199–203	2	190–265

*Abbreviation*: *N*_*a*_, the number of alleles

### Phylogenetic analysis

**Fig. 4:**
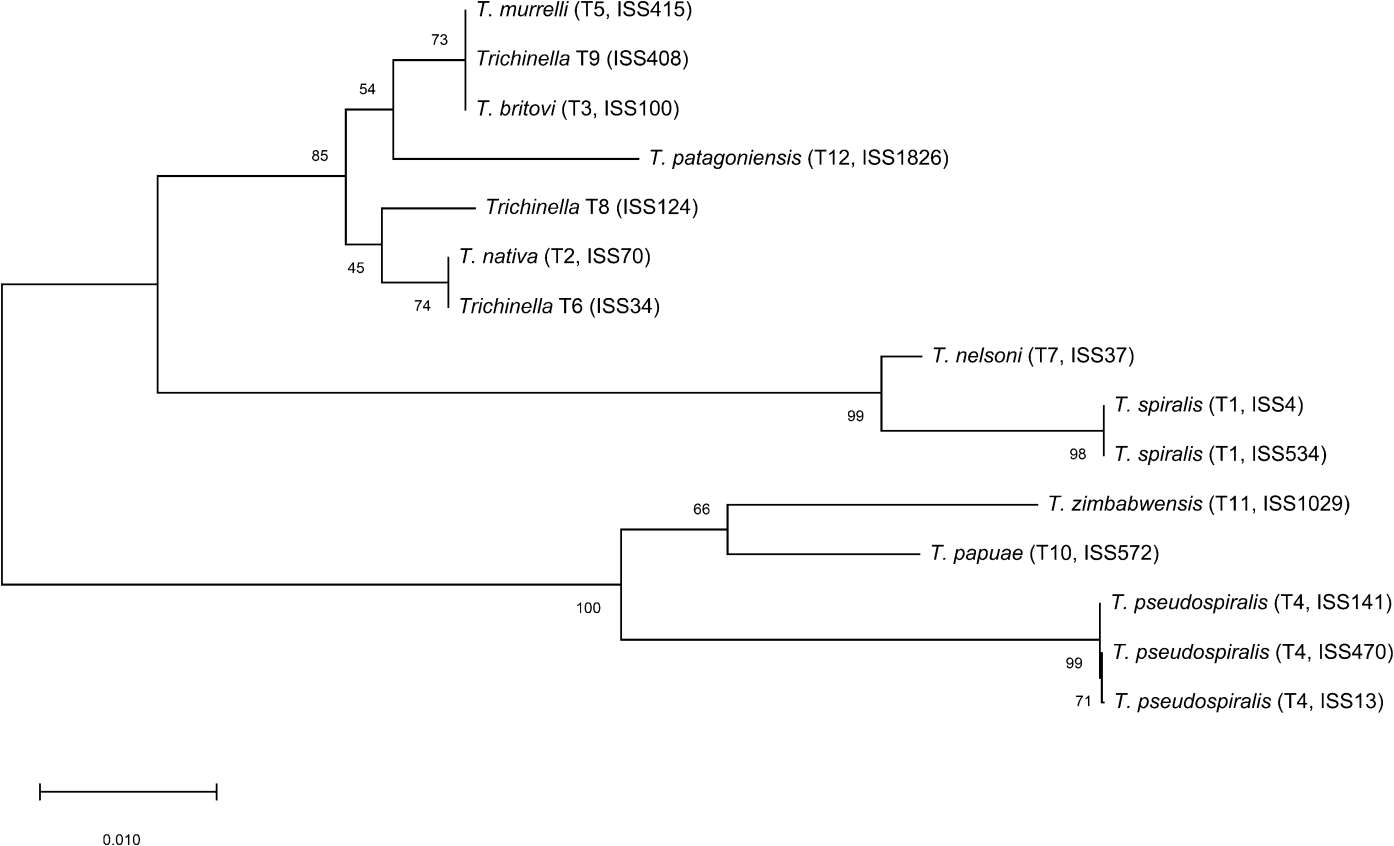
Phylogenetic tree inferred from the locus TsMs03 sequences of international standard strains of *Trichinella* spp. Primary phylogenetic analysis showed that 12 *Trichinella* species/genotypes clustered into two groups: encapsulated and non-encapsulated larvae group

Primary phylogenetic analysis showed that all *Trichinella* spp. clustered into two clades: encapsulated larvae and non-encapsulated larvae group (Fig. 4). Sister relationship was observed for *T. spiralis* and *T. nelsoni* in comparison to other species with encapsulated larvae. *Trichinella papuae* and *T. zimbabwensis* were more closely related to each other than to *T. pseudospiralis*.

## Discussion

Microsatellites have been used in genetic diversity and genetic mapping studies in various organisms [33–35], partly because of their high polymorphism and the ability to detect alleles at a given locus in individual organisms [36, 37]. In previous studies, most of microsatellites in *T. spiralis* were designed based on expressed sequence tag (EST) databases [20–22]. The present study identified 93,140 microsatellites in the *T. spiralis* genomes using MISA, which accounted for 2.25% of the total genome sequence. The relative abundance of microsatellite sequences was estimated at 1.591 loci per kb of the *T. spiralis* genomes.

Generally, microsatellites decrease in abundance with increasing repeat length [38, 39], and this trend has been observed in many organisms [40]. Previous comparative studies of microsatellites from eukaryotic genomes have found that the composition characteristics and distribution patterns significantly varied by species [39, 41]. *Caenorhabditis elegans* has a low frequency of microsatellites in its genome, even lower than *Saccharomyces cerevisiae* and other fungi [19, 42, 43]. In general, eukaryotic genomes are characterized by the prevalence of mononucleotide repeat motifs [19, 44]. For instance, mononucleotide repeats are the most abundant class of microsatellites in *C. elegans* [19] and *Meloidogyne incognita* [45]. However, dinucleotide repeats are the most abundant type of motif in rodents [19] and most dicot plant species [46]. Moreover, trinucleotide repeats are dominant in some algae and fungi species [44, 47], potentially indicating their genomic structural similarity with prokaryotes [48]. In contrast, tetra- to hexanucleotide repeats are less abundant in eukaryotic genomes [49, 50]. Intriguingly, our results suggested a different distribution pattern for *T. spiralis*: hexa- > tri- > tetra- > di- > penta- > mononucleotide repeats. The repeat frequency of hexanucleotides (49.51%) was higher than other repeat classes. This may be a characteristic that is unique to *T. spiralis.* It is also possible that the abundance of repeats is influenced by secondary structures and DNA replication [49].

Among mononucleotide repeats, the motif (A/T)_n_ is predominant, while (C/G)_n_ repeats are rare [45, 48]. Our results for the most dominant motif type in mono- to hexanucleotide repeat classes of *T. spiralis* showed similar (A+T)-rich motif patterns, where A/T, AT, AAT, AAAT, AAAAT, and AAAAAT were the predominant repeats. The possible reasons for this (A + T)-rich motif pattern may be as follows: (A + T)-rich motifs can decrease the annealing temperature and accelerate strand separation, and the AT content increases through DNA replication and slippage [49]. Secondly, DNA methylation can generate regions with high mutagenic rates, where the cytidine monophosphate becomes transformed into thymine. This type of mutation results from the deamination of methylation sites, leading to a combination of (A + T)-rich repeats. DNA methylation has been confirmed in the three life-cycle stages of *T. spiralis*, making it the only nematode species known to date with epigenetic modification of its genome [51]. In addition, these repeats may be favored because the order of bases can directly influence chromatin structure, protein coding and gene function [50].

Previous studies have shown that *Trichinella* spp. are considered to have low intraspecific genetic diversity and genetic differentiation between populations [6, 21, 52–58]. The unique life-cycle of *Trichinella* species can often promote sibling inbreeding and reduced population size [58]. Therefore, successful selection of microsatellite markers with relatively high abundance and polymorphism might be very difficult. Although the microsatellites of *T. spiralis* were detected in 12% of the 1000 EST sequences by La Rosa et al. [21], only seven microsatellite markers were suitable for genetic subgroup analysis. In the present study, 16 microsatellite markers with high polymorphism were selected and identified from 1000 candidate microsatellite loci.

To verify microsatellite markers with high polymorphism, we ranked the informativeness of markers using 120 individuals into highly (PIC > 0.50), reasonably (PIC of 0.25–0.50) and slightly informative (PIC < 0.25), as proposed by Botstein et al. [59]. Sixteen markers with high PIC were selected in 12 isolates of *T. spiralis* in China. The number of alleles per locus were positively correlated with the length of the repeat region, such as the locus TsMs03, which had the highest number of alleles and the longest repeat sequence (TAATT)_17_. Previous studies have shown that long loci have higher mutation rates than short loci [36, 60]. The HWE describes how allele and genotype frequencies are related. Deviations often occur in the presence of small sample size, inbreeding, or the effects of population subdivision [61]. Unfortunately, however, four microsatellite sites in tested populations deviated significantly from HWE after Bonferroni correction (*P* < 0.003) [62]. In addition, *H*_*O*_ was much lower than *H*_*E*_ in these 16 loci, which led to the observation of limited polymorphism to some extent.

Zarlenga et al. [63] found that *T. spiralis* diverged early in the genus *Trichinella*. An analysis of population variability used nine microsatellite markers and observed more allelic richness among eight isolates originating in Asia compared to the remaining isolates from Europe, North Africa, and North and South America, suggested that *T. spiralis* populations are more diverse in East Asia, where pigs were first domesticated [20]. Hence, in this study, we developed microsatellite loci and selected the ones with high polymorphism in 12 isolates of *T. spiralis* in China. The flanking sequences of the selected loci were relatively conserved in other *Trichinella* spp. Thus, ten of the 16 loci were amplified successfully in all 12 *Trichinella* spp. Therefore, the microsatellite loci developed in this study are good candidate loci to study the genetic variation and structure of *Trichinella* spp. beyond *T. spiralis*. Two loci, TsMs07 and TsMs08, were successfully amplified from all *Trichinella* spp., except for *T. pseudospiralis*. Recent studies have indicated that all five geographical isolates of *T. pseudospiralis* had one geographical origin that might diverge from *T. papuae* and *T. zimbabwensis*. Taken together, our results were consistent with other studies that *T. papuae* and *T. zimbabwensis* appeared to be basal in the group of species with non-encapsulated larvae and *T. pseudospiralis* the most recently evolved. The microsatellite analyses confirmed relationships among *Trichinella* spp. with non-encapsulated larvae, showing the utility of the new markers for investigating distantly related species within the genus [64].

## Conclusions

We reported the identification of microsatellite sequences from the genome sequence data of *T. spiralis* with MISA. Among them, 16 microsatellites with high polymorphisms among 12 isolates of *T. spiralis* from various geographical regions in China were identified, and 10 microsatellites could be amplified successfully from all 12 *Trichinella* spp. The primary phylogenetic analysis suggested that the newly selected microsatellite markers could be applied to the analysis of genetic relationship of *Trichinella* spp. These microsatellite markers might serve as an important resource for the further study of *Trichinella* spp.

## Data Availability

The datasets supporting the findings of this article are included within the article.

## Abbreviations

MISA: MIcroSAtellite Identification Tool
*H*_*O*_: Observed heterozygosity
*H*_*E*_: Expected heterozygosity
PIC: Polymorphism information content
RFLP: Restriction fragment length polymorphism
SSCP: Single-strand conformation polymorphism
SSR: Simple sequence repeat
CEA: Capillary electrophoresis analysis
*N*_*a*_: Number of alleles
*N*_*e*_: Effective number of alleles
HWE: Hardy–Weinberg equilibrium
EST: Expressed sequence tag
